# Predictors of micro-costing components in liver transplantation

**DOI:** 10.6061/clinics/2017(06)02

**Published:** 2017-06

**Authors:** Luciana Bertocco de Paiva Haddad, Liliana Ducatti, Luana Regina Baratelli Carelli Mendes, Wellington Andraus, Luiz Augusto Carneiro D’Albuquerque

**Affiliations:** Divisao de Transplante de Figado e Orgaos do Aparelho Digestivo, Departamento de Gastroenterologia, Faculdade de Medicina, Universidade de São Paulo, Sao Paulo, SP, BR

**Keywords:** Liver Transplantation, Healthcare Costs, Complications

## Abstract

**OBJECTIVES:**

Although liver transplantation procedures are common and highly expensive, their cost structure is still poorly understood. This study aimed to develop models of micro-costs among patients undergoing liver transplantation procedures while comparing the role of individual clinical predictors using tree regression models.

**METHODS:**

We prospectively collected micro-cost data from patients undergoing liver transplantation in a tertiary academic center. Data collection was conducted using an Intranet registry integrated into the institution’s database for the storing of financial and clinical data for transplantation cases.

**RESULTS:**

A total of 278 patients were included and accounted for 300 procedures. When evaluating specific costs for the operating room, intensive care unit and ward, we found that in all of the sectors but the ward, human resources were responsible for the highest costs. High cost supplies were important drivers for the operating room, whereas drugs were among the top four drivers for all sectors. When evaluating the predictors of total cost, a MELD score greater than 30 was the most important predictor of high cost, followed by a Donor Risk Index greater than 1.8.

**CONCLUSION:**

By focusing on the highest cost drivers and predictors, hospitals can initiate programs to reduce cost while maintaining high quality care standards.

## INTRODUCTION

More than 6,000 liver transplant procedures are performed in the United States every year 1. Despite being common, liver transplants are among the most expensive procedures in abdominal surgery 2, and the sources of these costs are still poorly understood, especially in developing countries. Specifically, and to our knowledge, no previous articles have attempted to use prospectively collected micro-costing data to predict individual patient costs while also using tree regression models to evaluate the contribution of individual factors in Latin America.

The average cost for liver transplantation has been estimated at Can $89,066 and ranges from Can $30,505 to Can $690,431 3, although this cost structure varies across countries. For example, a recent systematic review found a major cost difference between the United States and other OECD (Organization for Economic Co-operation and Development) countries, with values of US $163,438 (US $145,277-181,598) and US $103,548 (US $85,514-$121,582) 4, respectively. Regardless of the underlying variation across countries, liver transplantation is an expensive procedure 5. The sources of these high costs are not well understood, with some authors reporting that transplant admission charges alone represent as much as 50% of the total cost 6. When accounting for the total cost, it has also been reported that the total hospital costs and reimbursements are substantially increased when patients have additional complications 7. Furthermore, the highest expenses for this procedure vary widely as a function of the underlying etiology, e.g., substantial differences exist between patients with a diagnosis of hepatitis B and those with hepatocellular carcinoma 8.

Accurately predicting individual patient cost is important because transplantations are expensive procedures with significant cost variation. Some authors have suggested simplified formulas for the overall procedure cost. Brown et al. proposed that final charges (US $) for liver transplants could be calculated through the following formula: 3,407 × blood urea nitrogen + 74,474 × UNOS (United Network for Organ Sharing) status 1 + 102,662. Importantly, blood urea nitrogen and UNOS status are the only variables in this equation 9. In another model, Axelrod predicted that the higher the patient’s MELD (Model for End-Stage Liver Disease) score, the more the patient would be charged for the surgical procedure 10. In addition, having a severe liver disease, cytomegalovirus infection, additional operative procedures, and biliary complications are all predictors of increased cost 3. Other models have focused on utilization metrics as the primary predictors, using simple formulas in which the duration of the pre-transplant stay in the intensive care unit, age, body mass index, and calculated MELD scores are all used to predict cost after transplantation 11. Finally, some models have attempted to predict costs using variables such as graft type, height, race, hepatic artery thrombosis, early allograft rejection, and participation in a transition to home program 12. Since these previous attempts to predict cost are all based on traditional modeling techniques, the final estimation is limited by the accuracy of the individual variables.

To address this gap, the objective of this study was to develop a series of predictive models that permit an evaluation of individual clinical variables in the prediction of different cost components.

## METHODS

Our objective was to develop a series of predictive models for liver transplantation micro-costs based on a prospective registry with consecutive patients. Our modeling strategy is based on the TRIPOD statement 13.

### Ethics

Approval was obtained from the Institutional Review Board of the School of Medicine of the University of São Paulo (HCFMUSP).

### Setting

All of the data were collected from patients visiting a single, tertiary, outpatient clinic at the University of São Paulo. These patients were followed throughout the entire care pathway, and the data collected included intra-operative data. Data collection was conducted using an Intranet registry that was integrated into the institution’s administrative data collection system. These systems are used for the storage of financial and clinical information for transplantation cases because the hospital receives funding from the National Health System (NHS) for transplant procedures. Registration, consultations, hospitalizations, medications and the materials used were all tracked for liver transplantation cases. In addition, a dedicated research staff was responsible for collecting a detailed list of material consumption, equipment, and medications, among other cost items. A debit card was used for all purchases; therefore, all price-related information was available. Individual patients’ expenses for all components were prospectively calculated during their in-hospital period and then updated daily. Participant accrual occurred between January 2012 and December 2014. The team performing the transplantation was the same throughout the study period.

### Participants

We included all adult patients above the age of 18 undergoing liver transplantation. These cases included standard, living donor, combined transplantation (liver and kidney) and re-transplantation. All of the procedures were conducted at our center.

### Outcomes

All of the costs from enrollment to transplantation and the post-transplantation period (in-hospital period) were captured in real time. We measured costs related to provider visits, pre-operative tests, management of comorbidities and complications, hospitalizations, and general costs during the post-transplant period. These data were categorized as total cost, margins, revenue, and costs specific to the operating room (laboratory, gases, equipment, blood services, materials, drugs, human resources, high-end supplies, and standard equipment), the ICU (laboratory, dialysis, endoscopy, per diem, equipment, imaging, blood services, gases, materials, drugs, food, human resources, and intensive care unit), and the ward (pathology, gases, equipment, dialysis, per diem, electricity, phone charges, cleaning services, infrastructure and administrative support, endoscopy, imaging, laboratory, blood services, materials, drugs, food, and human resources). The costs of human resources (doctors, nurses, nursing assistants, physical therapy, nutritionists, social workers and psychologists) were calculated as the cost time per professional, and the time was defined as the hours dedicated by the professional for each specific liver transplant patient. The costs were converted from Brazilian reais to US dollars at a 3.69 rate (2015-11-04).

### Predictors

After a detailed review of the available evidence on predictors of the cost of liver transplantation, we used clinical judgment and the previous literature to select our variables 6,14. Specifically, we selected the following variables: length of stay (intensive care unit, ward, and total) and clinical predictors such as age, gender, body mass index, transplant type (standard, living donor, re-transplantation, or combined transplantation), MELD score, liver disease etiology (viral hepatitis, alcoholic cirrhosis, primary sclerosing cholangitis, and hepatocellular carcinoma), liver disease complications, comorbidities (diabetes, hypertension, and renal failure among others) and post-operative complications (rejection, kidney failure, and infection), outcomes (discharge, death or re-transplant), and cause of death (shock and septicemia).

### Data analysis

We started the analysis by performing a graphical exploratory analysis evaluating frequency, percentage and near-zero variance for categorical variables, distribution for numeric variables, and missing values and patterns across all variables 15. Median values were used for stratification to enable a similar frequency across comparison groups. Patients were excluded from the analyses when missing values were required. For example, when stratifying an analysis by the MELD score, patients with missing values for the MELD score were excluded. This exclusion accounts for the inequality in the total number of samples in all of the tables.

We used linear regression models for the analysis. All of the results were reported as predicted medians with 95% confidence intervals. Tree regression pruning was based on the following algorithm: at each pair of nodes from a common parent, we assessed the error based on the testing data and specifically evaluated whether the sum of squares would decrease if the two nodes were removed. In the case of a positive answer, the nodes were removed; otherwise, they were left intact. Although tree regression models represent the best cut-points for the values predicting the outcomes, in contrast with linear regression models, their results cannot be represented in a single equation. However, these models have a graphical representation that we present along with the interpretation of our results. All of the analyses were performed with the R statistical language and using regression trees based on the rpart package.

## RESULTS

A total of 278 patients were included and accounted for 300 procedures. Most patients were male (63.7%) with a mean age of 52.32 +/- 13.68 years and a body mass index of 25.89 +/- 5.96. When evaluating the procedures (Table 1), most of them were standard transplants (78.1%) with a mean Donor Risk Index (DRI) of 1.43 (+/- 0.31). The average length of stay was close to 21 days, with more than 9 days spent in the intensive care unit. One-fifth of our subjects died after the procedure, and approximately ten percent of all patients had to undergo a re-transplant. MELD scores greater than the median value in this sample (cut point at 30) were associated with higher rates of mortality and total costs.

When evaluating specific costs for the operating room, intensive care unit and ward (transplant unit floor), we found that in all sectors but the ward, human resources were responsible for the highest costs. High cost supplies were important drivers for the operating room, whereas drugs were among the top four drivers for all sectors. Higher MELD scores were associated with increased costs for high-cost supplies, blood services and labs in the operating room, and several types of costs occurred in the intensive care unit and ward (Table 2).

When comparing specific components of the total, predicted, and adjusted cost in patients with high MELD scores, patients with MELD functional scores greater than 30 presented significantly higher total costs (Table 3).

**Figure 1:**
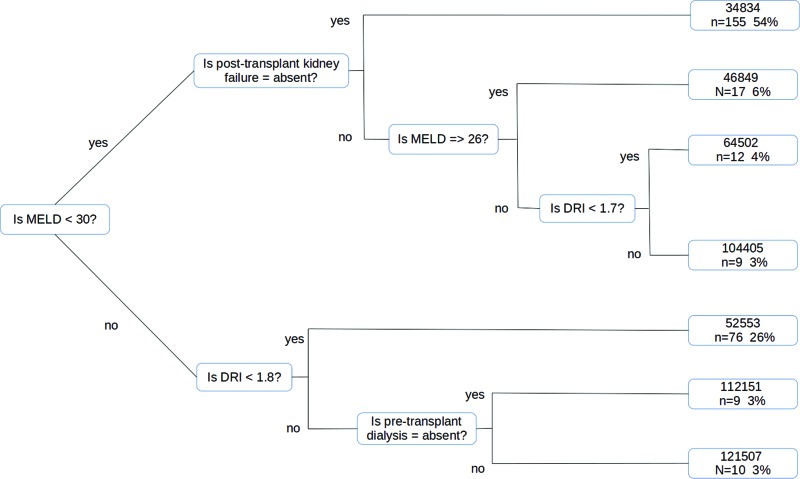
Tree regression model with individual predictors of total cost.

We then evaluated specific predictors of total costs associated with all baseline information, etiology, comorbidities, and complications. We found that a MELD score greater than 30 was the single most important predictor of costs, with the highest costs accompanying a MELD score greater than 30 and coupled to a DRI greater than 1.8 (Figure 1). This regression tree can be interpreted by following the patient characteristics from left to right. For example, if the MELD was less than 30 and if post-transplant kidney failure is absent, then the patient has a 54% chance of having costs among the top 50^th^ percentile.

As demonstrated in our tables in the appendix, when evaluating the total costs with respect to specific conditions, patients with hepatocellular carcinoma were associated with lower operating room, intensive care unit and ward costs; patients with primary sclerosing cholangitis were associated with lower intensive care unit costs; and patients with portal vein thrombosis were associated with higher total costs. In addition, death was also associated with an increased total cost of transplantation. Finally, linear regression modeling demonstrated that the time spent in the operating room was significantly associated with higher transplantation costs (*p*<0.001), whereas no statistically significant associations were found between costs, MELD score (*p*=0.724), and DRI (*p*=0.351).

## DISCUSSION

To the best of our knowledge, this is the first study utilizing tree regression models to focus on individual cost drivers after a liver transplant. When evaluating specific costs for the operating room, intensive care unit, and ward (transplantation unit floor), we found that in all sectors but the ward, human resources were responsible for the highest costs. High-cost supplies were important drivers for the operating room, whereas drugs were among the top four drivers for all sectors. When evaluating the predictors of total cost, a MELD score greater than 30 was the most important predictor of cost, with the highest costs accompanying a MELD greater than 30 and coupled with a DRI greater than 1.8.

To understand the cost structure related to liver transplants in Brazil, it is important to provide a context regarding the organ allocation system. Specifically, organs are allocated in Brazil based on the MELD score and blood type compatibility. Patients with hepatocellular carcinoma, refractory ascites, disabling encephalopathy, recurrent cholangitis and other specific diagnoses receive special attention and are moved to the top of the transplantation list. Others are followed up in relation to the functional MELD score for placement on the list. As a result, patients undergoing liver transplantation might be sicker than those in other countries as a function of our higher MELD scores at the time of transplant, which leads to significantly greater resource utilization and transplantation cost 6,16,17. Among the available healthcare cost studies evaluating liver transplants, there has been considerable variation in study design, financial aspects of transplantation, and the time period for the cost analysis. For example, the mean cost of liver transplantation in Europe is lower than in the United States, with the following rates reported: Italy, 77,475 euros 18; France, 850,515 euros 19; Netherlands, 107,0675 euros for chronic liver disease and 900,792 euros for acute liver failure 20; Finland, 141,768 euros 17; Germany, 52,570 euros 21; Switzerland, 118,457 euros; the United Kingdom, 49,920-70,200 euros; and the United States, 156,000-217,674 euros 22. In contrast, the total cost in Brazil is US$ 20,605.01 23.

It has been previously demonstrated that complications following liver transplantation significantly increase hospital costs 7,24,25. As a consequence, the prediction of liver transplantation costs is closely connected to the prediction of complications. The severity of liver disease, post-operative infections, such as cytomegalovirus infections, and complications involving the biliary tract have all been demonstrated to contribute to a rise in surgical costs 3. Specifically, in the pediatric population, complications such as acute cellular rejection, acute renal failure, hepatic artery thrombosis, and infections such as pneumonia accounted for a substantial increase in hospital costs and, consequently, a decrease in profit margins 26. However, liver transplants are not the only procedures with complications contributing to a rise in costs, and others include pancreas and kidney transplants 27. Although many of these complications are unpredictable, we currently know that high MELD scores and previous renal insufficiency present higher risks of post-transplant complications 28,29. As expected, when complications develop, the corresponding length of the hospital stay also increases.

The length of the hospital stay is often cited as an important predictor of cost after liver transplantation. In general, the greater the number of days spent in the hospital after liver transplant the higher the cost of the overall transplantation 30–32. It has been previously demonstrated that the length of the hospital stay is one of the main determinants of overall hospital fees for surgical patients 33. However, as some of our models have shown, the impact of the length of stay on cost is increased by the presence of comorbidities, with longer stay intervals sometimes only generating a negligible impact on total hospital costs 34,35. Overall, the interplay of multiple determinants of cost is important, and comorbidities play a particularly important role.

Comorbidities, such as viral hepatitis, advanced liver disease, and portal thrombosis, have all been associated with higher costs of liver transplant 18,30, and impaired renal function is one of its main determinants 9,18. Liver transplants are not isolated in this aspect in that comorbidities have also been shown to increase the cost of kidney transplantation 36. Several authors have used comorbidity indices to combine different factors in the prediction of outcomes after liver transplantation. For example, Wasilewicz et al. adopted the Charlson comorbidity index 37 to predict survival after liver transplantation 38. The Charlson index has also been used to predict health care economic endpoints including cost and resource utilization 39.

Despite filling an important gap in the literature, our study does have limitations that are mostly associated with its observational design. First, our cost measures were not unanimously validated by different observers and thus introduced a potential classification bias. Second, we did not include self-reported measures of quality of life or dysfunction, which could later be used in cost-utility analyses. These measures constitute an important metric in that they take into account a direct patient perspective, which is clearly missing when only cost-driven measurements are used. This absence in our study was primarily driven by logistical reasons in that the inclusion of self-reported questionnaires would significantly increase the complexity of data collection. Third, despite our best efforts in controlling for missing rates, some of our variables, such as the DRI, presented particularly high rates. To minimize this limitation, we utilized imputation algorithms followed by sensitivity analyses to ensure that our final conclusions were valid under different assumptions. Fourth, given that our sample was not randomly drawn from a larger patient population, its external validity can be questioned. Although future studies should certainly aim for larger and more representative samples, our sample is by no means atypical for its setting, which makes our conclusions valid for similar populations globally. Fifth, because the same team performed the transplants throughout the study period, we cannot evaluate the impact of trainees on cost. In addition, because we do not have the breakdown data with respect to ventilator time in the intensive care unit, we cannot further investigate the underlying reasons behind its cost. Finally, several other statistical approaches could have been used, including machine learning, which allow for greater predictive performance and Bayesian Network models that might in turn allow for causal inferences. However, the former usually requires larger samples, while the latter is less familiar to clinical audiences and could therefore lead to confusion in the interpretation of our results.

In conclusion, our study holds promise in that cost data can be used to assess future areas that can lead to cost-saving strategies while maintaining good quality of care 25,27,40–42. Cost studies are particularly relevant for quality assurance and safety programs because the improvement of quality must be conducted within an environment that takes health economics into account. This is especially important in countries undergoing cost containment, such as developing countries and even the United States under escalating cost increases and the Affordable Care Act. We therefore recommend that healthcare institutions should undergo continuous cost evaluations to determine their main cost drivers and to determine cost predictors for liver transplantation. Such studies will allow institutions and public health systems to predict the cost of each procedure using pre-transplant data from the recipient and donor.

## AUTHOR CONTRIBUTIONS

Haddad LB, Mendes LR, Andraus W and D'Albuquerque LC were responsible for the study conception and design. Haddad LB, Ducatti L, Mendes L and Andraus W were responsible for the analysis and interpretation. Haddad LB, Ducatti L, Andraus W and D'Albuquerque LC were responsible for the manuscript drafting. Haddad LB and D'Albuquerque LC were responsible for the manuscript review. Haddad LB was responsible for the final approval of the manuscript.

## Figures and Tables

**Table 1:** General characteristics of transplant procedures stratified by the median MELD score.

Variable [Missing]	Total (278)	MELD Functional < 30 (179)	MELD Functional >= 30 (99)	*p*
Male [0]	177 (63.7%)	124 (69.3%)	53 (53.5%)	0.013
Age [0]	52.32 (+- 13.68)	55.68 (+- 12.32)	46.25 (+- 13.99)	<0.001
BMI [3]	25.89 (+- 5.96)	25.66 (+- 5.77)	26.3 (+- 6.31)	0.406
Transplant type [0]				0.002
- Standard	217 (78.1%)	143 (79.9%)	74 (74.7%)	
- Re-transplant	35 (12.6%)	14 (7.8%)	21 (21.2%)	
- Living donor	14 (5%)	13 (7.3%)	1 (1%)	
- Double transplant	12 (4.3%)	9 (5%)	3 (3%)	
Donor risk index [130]	1.43 (+- 0.31)	1.43 (+- 0.31)	1.44 (+- 0.32)	0.904
Total length of stay [0]	21.39 (+- 25.07)	19.24 (+- 17.32)	25.27 (+- 34.75)	0.108
Length of stay at ICU [0]	9.38 (+- 12.39)	7.61 (+- 9.18)	12.58 (+- 16.27)	0.006
Length of stay at the ward [0]	11.57 (+- 15.78)	11.63 (+- 12.61)	11.45 (+- 20.38)	0.938
Outcome [5]				<0.001
- Discharge	181 (66.3%)	128 (72.7%)	53 (54.6%)	
- Death	64 (23.4%)	25 (14.2%)	39 (40.2%)	
- Re-transplant	28 (10.3%)	23 (13.1%)	5 (5.2%)	
Total cost [0]	13613.77 (+- 12293.95)	11542.2 (+- 7891.81)	17359.33 (+- 17090.13)	0.002

**Table 2:** Specific costs for the operating room, intensive care unit and ward stratified by the median MELD score.

Variable [Missing]	Total (278)	MELD Functional < 30 (179)	MELD Functional >= 30 (99)	*p*
Operating room cost [0]	5013.18 (+- 1169.68)	4919.48 (+- 1054.35)	5182.59 (+- 1342.73)	0.094
- Human resources [0]	1481.77 (+- 404.22)	1514.28 (+- 432.86)	1422.99 (+- 340.71)	0.054
- High-cost supplies [0]	1451.69 (+- 264.66)	1477.98 (+- 222.4)	1404.14 (+- 323.41)	0.045
- Drugs [0]	930.91 (+- 506.62)	898.57 (+- 465.91)	989.37 (+- 570.81)	0.178
- Materials [0]	466.99 (+- 449.65)	444.11 (+- 453.42)	508.38 (+- 442.02)	0.251
- Blood services [0]	279.47 (+- 456.23)	193.45 (+- 403.5)	435 (+- 504.63)	<0.001
- Equipment [0]	168.2 (+- 45.88)	171.89 (+- 49.14)	161.53 (+- 38.68)	0.054
- Labs [0]	93.02 (+- 135.55)	70.54 (+- 96.63)	133.65 (+- 179.95)	0.002
- Gases [0]	23.65 (+- 6.45)	24.17 (+- 6.91)	22.71 (+- 5.44)	0.054
- Room [0]	85.41 (+- 23.3)	87.29 (+- 24.95)	82.03 (+- 19.64)	0.054
- Flowmeter [0]	36 (+- 15.22)	37.2 (+- 13.96)	33.84 (+- 17.11)	0.097
- Hours [0]	9.71 (+- 2.64)	9.91 (+- 2.83)	9.33 (+- 2.23)	0.062
Intensive care unit cost [0]	5792.53 (+- 9508.94)	4145.58 (+- 6381.22)	8770.35 (+- 12949.98)	0.001
- Human resources [0]	2071.29 (+- 2903.29)	1626.99 (+- 2045.01)	2874.63 (+- 3900.85)	0.004
- Dialysis [0]	950.68 (+- 3266.94)	562.42 (+- 2060.89)	1652.67 (+- 4656.15)	0.029
- Drugs [0]	859.47 (+- 2751.78)	572.01 (+- 1880.11)	1379.22 (+- 3815.64)	0.05
- Labs [0]	638.35 (+- 881.47)	426.13 (+- 528.91)	1022.06 (+- 1207.1)	<0.001
- Materials [0]	387.67 (+- 571.33)	302.71 (+- 392.33)	541.28 (+- 778.48)	0.005
- Blood services [0]	344.33 (+- 695.75)	257.99 (+- 658.21)	500.43 (+- 736.95)	0.007
- Imaging [0]	177.41 (+- 318.25)	116.59 (+- 202.77)	287.4 (+- 438.88)	<0.001
- Equipment [0]	156.11 (+- 218.82)	122.62 (+- 154.13)	216.66 (+- 294.01)	0.004
- Gases [0]	111.95 (+- 156.92)	87.94 (+- 110.53)	155.37 (+- 210.84)	0.004
- Food [0]	68.91 (+- 167.84)	51.38 (+- 143.16)	100.6 (+- 202.05)	0.034
- Per diem cost deprecated [0]	22.45 (+- 31.46)	17.63 (+- 22.16)	31.15 (+- 42.27)	0.004
- Endoscopy [0]	3.91 (+- 32.02)	1.17 (+- 5.02)	8.87 (+- 53.04)	0.153
Ward cost [0]	2808.06 (+- 3820.61)	2477.14 (+- 2640.44)	3406.39 (+- 5294.72)	0.104
- Labs [0]	701.23 (+- 990.82)	588.58 (+- 631.39)	904.9 (+- 1409.14)	0.036
- Human resources [0]	633.78 (+- 849.36)	638.06 (+- 685.22)	626.05 (+- 1089.11)	0.921
- Materials [0]	544.13 (+- 851.38)	511.67 (+- 663.38)	602.84 (+- 1115.39)	0.458
- Drugs [0]	443.79 (+- 1085.53)	319.58 (+- 658.07)	668.37 (+- 1570.05)	0.037
- Dialysis [0]	127.82 (+- 1156.92)	102.77 (+- 1144.93)	173.11 (+- 1182.79)	0.632
- Anatomic pathology [0]	118.76 (+- 89.37)	110.55 (+- 89.29)	133.59 (+- 88.01)	0.039
- Imaging [0]	103.82 (+- 188.16)	82.72 (+- 137.06)	141.96 (+- 252.32)	0.032
- Food [0]	89.79 (+- 197.02)	77.18 (+- 140.16)	112.6 (+- 270.59)	0.227
- Blood services [0]	42.69 (+- 166.77)	17.56 (+- 66.94)	88.12 (+- 259.3)	0.009
- Per diem cost deprecated [0]	19.77 (+- 26.5)	19.91 (+- 21.38)	19.53 (+- 33.98)	0.921
- Endoscopy [0]	6.49 (+- 44.13)	4.72 (+- 38.81)	9.69 (+- 52.47)	0.409
- Equipment [0]	6.44 (+- 36.18)	3.64 (+- 24.57)	11.5 (+- 50.63)	0.149
- Gases [0]	2.23 (+- 30.65)	0.2 (+- 1.78)	5.91 (+- 51.27)	0.271
Total lab cost [0]	1367.11 (+- 1609.41)	1040.91 (+- 966.04)	1956.9 (+- 2253.98)	<0.001
OR average medical surgical center cost [0]	78.52 (+- 172.2)	91.47 (+- 182.81)	55.13 (+- 149.19)	0.074

**Table 3:** Predicted and adjusted median costs and 95 % confidence intervals.

Cost Component	MELD Functional < 30	MELD Functional >= 30
Total length of stay	16.4 (12.96, 20.77)	16.37 (12.36, 21.68)
Operating room cost	5042.06 (4776.22, 5322.71)	5282.81 (4952.98, 5634.61)
Intensive care unit cost	3342.48 (2513.81, 4444.31)	5651.5 (4026.17, 7932.95)
Ward cost	995.76 (687.1, 1443.09)	1073.51 (690.27, 1669.52)
Total cost	11751.17 (10376.08, 13308.49)	15464.97 (13335.85, 17934)

**Table 4:** Total costs for the operating room, intensive care unit and ward stratified by hepatocellular carcinoma (HCC).

Variable [Missing]	Total (168)	Hepatocellular carcinoma absent (114)	Hepatocellular carcinoma present (54)	*p*
Operating room cost [0]	5483.51 (+- 941.72)	5583.58 (+- 1002.05)	5272.24 (+- 765.85)	0.028
- Human resources [0]	1470.64 (+- 388.09)	1479.69 (+- 412.76)	1451.54 (+- 332.84)	0.637
- High-cost supplies [0]	1397.31 (+- 329.64)	1356.52 (+- 366.04)	1483.43 (+- 213.51)	0.005
- Drugs [0]	1163.68 (+- 360.56)	1179.29 (+- 393.8)	1130.72 (+- 278.13)	0.36
- Materials [0]	641.65 (+- 439.5)	688.32 (+- 481)	543.15 (+- 317.49)	0.021
- Blood services [0]	383.33 (+- 490.23)	449.83 (+- 515.23)	242.95 (+- 402.1)	0.005
- Equipment [0]	166.94 (+- 44.05)	167.96 (+- 46.85)	164.77 (+- 37.78)	0.637
- Labs [0]	126.41 (+- 161.8)	132.52 (+- 171.87)	113.5 (+- 138.76)	0.445
- Gases [0]	23.48 (+- 6.2)	23.62 (+- 6.59)	23.17 (+- 5.31)	0.637
- Room [0]	84.77 (+- 22.37)	85.29 (+- 23.79)	83.67 (+- 19.19)	0.637
- Flowmeter [0]	31.81 (+- 18.42)	30.13 (+- 19.32)	35.34 (+- 15.95)	0.068
- Hours [0]	9.64 (+- 2.54)	9.7 (+- 2.7)	9.5 (+- 2.18)	0.605
Intensive care unit cost [0]	6933 (+- 11206.92)	8286.71 (+- 12656.89)	4075.16 (+- 6475.2)	0.005
- Human resources [0]	2111.6 (+- 2997.94)	2476.36 (+- 3250.06)	1341.56 (+- 2215.48)	0.009
- Dialysis [0]	1179.06 (+- 3931.16)	1385.49 (+- 4457.92)	743.27 (+- 2457.4)	0.232
- Drugs [0]	1324.37 (+- 3448.97)	1715.37 (+- 4087.75)	498.92 (+- 912.32)	0.003
- Labs [0]	803.39 (+- 970.79)	925.79 (+- 1084.32)	545.01 (+- 602.06)	0.004
- Materials [0]	385.52 (+- 593.81)	448.22 (+- 652.63)	253.16 (+- 420.28)	0.021
- Blood services [0]	501.32 (+- 819.62)	589.8 (+- 882.11)	314.53 (+- 636.63)	0.023
- Imaging [0]	223.22 (+- 362.72)	265.97 (+- 413.13)	132.96 (+- 196.05)	0.005
- Equipment [0]	159.15 (+- 225.95)	186.64 (+- 244.96)	101.11 (+- 166.98)	0.009
- Gases [0]	114.13 (+- 162.04)	133.85 (+- 175.66)	72.51 (+- 119.75)	0.009
- Food [0]	104.33 (+- 207.05)	127.26 (+- 236.15)	55.92 (+- 112.54)	0.009
- Per diem cost deprecated [0]	22.88 (+- 32.49)	26.84 (+- 35.22)	14.54 (+- 24.01)	0.009
- Endoscopy [0]	4.01 (+- 30.25)	5.13 (+- 36.45)	1.66 (+- 6.54)	0.327
Ward cost [0]	3474.9 (+- 4573.5)	3909.41 (+- 5310.57)	2557.6 (+- 2124.99)	0.02
- Labs [0]	903.96 (+- 1146.54)	990.4 (+- 1272.35)	721.48 (+- 800.34)	0.098
- Human resources [0]	643.94 (+- 901.43)	711.36 (+- 1038.87)	501.59 (+- 478.42)	0.075
- Materials [0]	578.51 (+- 978.09)	610.98 (+- 1069.66)	509.96 (+- 753.56)	0.482
- Drugs [0]	666.86 (+- 1327.66)	773.55 (+- 1507.17)	441.64 (+- 796.76)	0.064
- Dialysis [0]	205 (+- 1483.91)	293.62 (+- 1796.56)	17.91 (+- 65.49)	0.105
- Anatomic pathology [0]	137.19 (+- 78.28)	146.62 (+- 88.66)	117.29 (+- 44.19)	0.005
- Imaging [0]	147.62 (+- 217.6)	175.55 (+- 249.42)	88.68 (+- 106.5)	0.002
- Food [0]	133.83 (+- 240.43)	152.23 (+- 282.82)	94.99 (+- 96.6)	0.055
- Blood services [0]	69.45 (+- 210.26)	87.34 (+- 243.03)	31.69 (+- 105.99)	0.04
- Per diem cost deprecated [0]	20.09 (+- 28.13)	22.2 (+- 32.42)	15.65 (+- 14.93)	0.075
- Endoscopy [0]	8.18 (+- 49.27)	11.47 (+- 59.5)	1.22 (+- 5.55)	0.071
- Equipment [0]	10.66 (+- 46.11)	8.48 (+- 44.12)	15.25 (+- 50.17)	0.398
- Gases [0]	3.7 (+- 39.41)	5.32 (+- 47.82)	0.27 (+- 0.76)	0.262
Total labs cost [0]	1752.91 (+- 1823.34)	1975.33 (+- 2063.57)	1283.36 (+- 1032.23)	0.004
OR average medical surgical center cost [0]	2.71 (+- 35.09)	3.99 (+- 42.59)	0 (+- 0)	0.319

**Table 5:** Total costs for the operating room, intensive care unit and ward stratified by primary sclerosing cholangitis (PSC).

Variable [Missing]	Total (168)	Primary sclerosing cholangitis absent (161)	Primary sclerosing cholangitis present (7)	*p*
Operating room cost [0]	5483.51 (+- 941.72)	5488.48 (+- 957.45)	5369.11 (+- 471.19)	0.554
- Human resources [0]	1470.64 (+- 388.09)	1466.25 (+- 390.07)	1571.59 (+- 349.7)	0.464
- High-cost supplies [0]	1397.31 (+- 329.64)	1420.02 (+- 305.54)	875.12 (+- 450.6)	0.018
- Drugs [0]	1163.68 (+- 360.56)	1164.39 (+- 367.86)	1147.39 (+- 98.25)	0.723
- Materials [0]	641.65 (+- 439.5)	621.46 (+- 419.67)	1106.07 (+- 646)	0.095
- Blood services [0]	383.33 (+- 490.23)	392.59 (+- 496.1)	170.56 (+- 266.48)	0.074
- Equipment [0]	166.94 (+- 44.05)	166.44 (+- 44.28)	178.4 (+- 39.69)	0.464
- Labs [0]	126.41 (+- 161.8)	123.55 (+- 161.13)	192.18 (+- 175.97)	0.347
- Gases [0]	23.48 (+- 6.2)	23.41 (+- 6.23)	25.09 (+- 5.58)	0.464
- Room [0]	84.77 (+- 22.37)	84.52 (+- 22.49)	90.59 (+- 20.16)	0.464
- Flowmeter [0]	31.81 (+- 18.42)	32.66 (+- 17.9)	12.12 (+- 20.69)	0.039
- Hours [0]	9.64 (+- 2.54)	9.61 (+- 2.55)	10.29 (+- 2.29)	0.472
Intensive care unit cost [0]	6933 (+- 11206.92)	7122.8 (+- 11406.97)	2567.62 (+- 1641.24)	<0.001
- Human resources [0]	2111.6 (+- 2997.94)	2172.47 (+- 3047.75)	711.69 (+- 264.13)	<0.001
- Dialysis [0]	1179.06 (+- 3931.16)	1230.33 (+- 4008.32)	0 (+- 0)	<0.001
- Drugs [0]	1324.37 (+- 3448.97)	1354.29 (+- 3514.3)	636.16 (+- 1082.42)	0.17
- Labs [0]	803.39 (+- 970.79)	815.89 (+- 989.6)	515.96 (+- 123.25)	0.002
- Materials [0]	385.52 (+- 593.81)	387.6 (+- 600.9)	337.66 (+- 427.27)	0.775
- Blood services [0]	501.32 (+- 819.62)	518.05 (+- 832.79)	116.46 (+- 151.07)	<0.001
- Imaging [0]	223.22 (+- 362.72)	227.44 (+- 369.37)	126.09 (+- 110.93)	0.068
- Equipment [0]	159.15 (+- 225.95)	163.74 (+- 229.71)	53.64 (+- 19.91)	<0.001
- Gases [0]	114.13 (+- 162.04)	117.42 (+- 164.73)	38.47 (+- 14.28)	<0.001
- Food [0]	104.33 (+- 207.05)	107.83 (+- 210.8)	23.77 (+- 18.2)	<0.001
- Per diem cost deprecated [0]	22.88 (+- 32.49)	23.54 (+- 33.02)	7.71 (+- 2.86)	<0.001
- Endoscopy [0]	4.01 (+- 30.25)	4.19 (+- 30.89)	0 (+- 0)	0.087
Ward cost [0]	3474.9 (+- 4573.5)	3491.11 (+- 4552.56)	3101.95 (+- 5415.87)	0.857
- Labs [0]	903.96 (+- 1146.54)	911.52 (+- 1155.3)	730 (+- 979.52)	0.649
- Human resources [0]	643.94 (+- 901.43)	660.56 (+- 914.84)	261.55 (+- 346.79)	0.023
- Materials [0]	578.51 (+- 978.09)	550.53 (+- 846.65)	1222.1 (+- 2647.15)	0.528
- Drugs [0]	666.86 (+- 1327.66)	676.64 (+- 1344.47)	441.89 (+- 892.77)	0.527
- Dialysis [0]	205 (+- 1483.91)	213.91 (+- 1515.39)	0 (+- 0)	0.075
- Anatomic pathology [0]	137.19 (+- 78.28)	138.32 (+- 79.37)	111.11 (+- 41.37)	0.144
- Imaging [0]	147.62 (+- 217.6)	149.09 (+- 219.71)	113.92 (+- 170.91)	0.615
- Food [0]	133.83 (+- 240.43)	134.96 (+- 242.13)	107.82 (+- 211.25)	0.751
- Blood services [0]	69.45 (+- 210.26)	67.99 (+- 210.26)	102.96 (+- 223.96)	0.698
- Per diem cost deprecated [0]	20.09 (+- 28.13)	20.61 (+- 28.55)	8.16 (+- 10.82)	0.023
- Endoscopy [0]	8.18 (+- 49.27)	8.5 (+- 50.32)	0.77 (+- 1.32)	0.055
- Equipment [0]	10.66 (+- 46.11)	11.06 (+- 47.06)	1.55 (+- 2.8)	0.015
- Gases [0]	3.7 (+- 39.41)	3.85 (+- 40.25)	0.12 (+- 0.27)	0.241
Total labs cost [0]	1752.91 (+- 1823.34)	1772.06 (+- 1851.72)	1312.53 (+- 927.96)	0.26
OR average medical surgical center cost [0]	2.71 (+- 35.09)	2.82 (+- 35.84)	0 (+- 0)	0.319

**Table 6:** Cost stratified by portal vein thrombosis (PVT).

Variable [Missing]	Total (300)	Portal vein thrombosis absent (283)	Portal vein thrombosis present (17)	*p*
Operating room cost [12]	4978.97 (+- 1167.67)	4950.58 (+- 1175.22)	5431.46 (+- 958.63)	0.063
- Human resources [12]	1488.68 (+- 407.86)	1486.78 (+- 398.37)	1518.95 (+- 552.15)	0.816
- High-cost supplies [12]	1454.57 (+- 260.45)	1463.17 (+- 247.36)	1317.45 (+- 403.77)	0.16
- Drugs [12]	914.38 (+- 505.31)	906.17 (+- 512.25)	1045.26 (+- 364.42)	0.153
- Materials [12]	450.78 (+- 449.97)	441.29 (+- 455.7)	601.97 (+- 318.92)	0.064
- Blood services [12]	269.76 (+- 451.13)	253.3 (+- 439.74)	532.26 (+- 556.46)	0.058
- Equipment [12]	168.99 (+- 46.3)	168.77 (+- 45.22)	172.42 (+- 62.68)	0.816
- Labs [12]	89.81 (+- 134.24)	89.24 (+- 137.32)	98.91 (+- 70.21)	0.615
- Gases [12]	23.76 (+- 6.51)	23.73 (+- 6.36)	24.25 (+- 8.82)	0.816
- Room [12]	85.81 (+- 23.51)	85.7 (+- 22.96)	87.56 (+- 31.83)	0.816
- Flowmeter [12]	36.23 (+- 14.99)	36.46 (+- 14.75)	32.43 (+- 18.54)	0.391
- Hours [12]	9.75 (+- 2.67)	9.74 (+- 2.61)	9.94 (+- 3.61)	0.822
Intensive care unit cost [12]	5601.05 (+- 9396.4)	5546.73 (+- 9370.92)	6466.91 (+- 10051.54)	0.718
- Human resources [12]	2006.58 (+- 2872.67)	1986.92 (+- 2826.92)	2319.98 (+- 3613.45)	0.714
- Dialysis [12]	917.67 (+- 3214.25)	908.12 (+- 3241.82)	1069.91 (+- 2819.12)	0.823
- Drugs [12]	829.63 (+- 2708.01)	841.6 (+- 2782.69)	638.76 (+- 911.32)	0.47
- Labs [12]	616.21 (+- 873.84)	607.71 (+- 876.75)	751.78 (+- 839.36)	0.502
- Materials [12]	375.61 (+- 564.89)	376.59 (+- 561.57)	360.05 (+- 633.92)	0.918
- Blood services [12]	332.37 (+- 686.43)	306.5 (+- 640.4)	744.78 (+- 1157.3)	0.141
- Imaging [12]	171.25 (+- 314.34)	172.11 (+- 319.26)	157.56 (+- 228.49)	0.807
- Equipment [12]	151.23 (+- 216.51)	149.75 (+- 213.06)	174.85 (+- 272.35)	0.714
- Gases [12]	108.46 (+- 155.27)	107.39 (+- 152.79)	125.4 (+- 195.31)	0.714
- Food [12]	66.52 (+- 165.38)	64.7 (+- 163.98)	95.52 (+- 189.46)	0.521
- Per diem cost deprecated [12]	21.74 (+- 31.13)	21.53 (+- 30.63)	25.14 (+- 39.16)	0.714
- Endoscopy [12]	3.77 (+- 31.46)	3.81 (+- 32.39)	3.18 (+- 7.29)	0.812
Ward cost [12]	2714 (+- 3786.2)	2767.54 (+- 3878.21)	1860.49 (+- 1583.63)	0.053
- Labs [12]	676.9 (+- 981.84)	688.96 (+- 1005.01)	484.73 (+- 453.71)	0.116
- Human resources [12]	613.65 (+- 841.18)	623.53 (+- 856.75)	456.13 (+- 526.96)	0.238
- Materials [12]	526.73 (+- 841.46)	533.87 (+- 860.12)	412.93 (+- 449.17)	0.327
- Drugs [12]	428.38 (+- 1069.56)	444.29 (+- 1099.02)	174.78 (+- 254.29)	0.004
- Dialysis [12]	123.38 (+- 1136.82)	131.12 (+- 1171.63)	0 (+- 0)	0.067
- Anatomic pathology [12]	114.63 (+- 90.46)	113.8 (+- 92.17)	127.86 (+- 56.74)	0.354
- Imaging [12]	100.21 (+- 185.83)	100.47 (+- 189.94)	96.08 (+- 102.88)	0.874
- Food [12]	86.68 (+- 194.26)	88.81 (+- 199.29)	52.61 (+- 73.07)	0.101
- Blood services [12]	41.21 (+- 164.03)	41.39 (+- 167.85)	38.25 (+- 84.63)	0.892
- Per diem cost deprecated [12]	19.15 (+- 26.25)	19.45 (+- 26.73)	14.23 (+- 16.44)	0.238
- Endoscopy [12]	6.26 (+- 43.37)	6.49 (+- 44.69)	2.61 (+- 4.92)	0.191
- Equipment [12]	6.22 (+- 35.56)	6.59 (+- 36.63)	0.25 (+- 0.92)	0.005
- Gases [12]	2.16 (+- 30.12)	2.29 (+- 31.04)	0.03 (+- 0.08)	0.232
Total labs cost [12]	1319.69 (+- 1600.83)	1322.7 (+- 1637.99)	1271.71 (+- 830.38)	0.822
OR average medical surgical center cost [12]	91.59 (+- 182.71)	97.34 (+- 186.87)	0 (+- 0)	<0.001

**Table 7:** Cost stratified by death.

Variable [Missing]	Total (261)	Alive (199)	Dead (62)	*p*
Operating room cost [0]	5089.3 (+- 1160.99)	4964.38 (+- 1082.93)	5490.26 (+- 1313.13)	0.005
- Human resources [0]	1479.35 (+- 404.54)	1452.69 (+- 334.99)	1564.91 (+- 568.81)	0.144
- High-cost supplies [0]	1446.28 (+- 272.29)	1432.64 (+- 290.3)	1490.05 (+- 199.77)	0.081
- Drugs [0]	961.92 (+- 507.6)	952.4 (+- 499.44)	992.48 (+- 535.99)	0.603
- Materials [0]	497.41 (+- 447.46)	509.98 (+- 477.41)	457.07 (+- 333.8)	0.331
- Blood services [0]	297.67 (+- 465.09)	218.53 (+- 372.54)	551.69 (+- 620.88)	<0.001
- Equipment [0]	167.93 (+- 45.92)	164.9 (+- 38.03)	177.64 (+- 64.57)	0.144
- Labs [0]	98.47 (+- 138.07)	96.43 (+- 129.06)	104.99 (+- 164.74)	0.709
- Gases [0]	23.61 (+- 6.46)	23.19 (+- 5.35)	24.98 (+- 9.08)	0.144
- Room [0]	85.28 (+- 23.32)	83.74 (+- 19.31)	90.21 (+- 32.79)	0.144
- Flowmeter [0]	35.59 (+- 15.61)	35.38 (+- 15.81)	36.25 (+- 15.06)	0.693
- Hours [0]	9.69 (+- 2.65)	9.51 (+- 2.19)	10.27 (+- 3.71)	0.127
Intensive care unit cost [0]	6047.92 (+- 9749.58)	5158.58 (+- 9138.43)	8902.42 (+- 11100.42)	0.018
- Human resources [0]	2119.51 (+- 2972.93)	1829.6 (+- 2410.3)	3050.05 (+- 4202.38)	0.033
- Dialysis [0]	1012.6 (+- 3362.71)	863.27 (+- 3407.62)	1491.88 (+- 3193.76)	0.186
- Drugs [0]	915.45 (+- 2831.25)	756.93 (+- 2708.06)	1424.24 (+- 3165)	0.138
- Labs [0]	673.74 (+- 896.9)	615.13 (+- 872.88)	861.86 (+- 953.09)	0.073
- Materials [0]	396.05 (+- 585.39)	357.5 (+- 516.21)	519.82 (+- 758.43)	0.119
- Blood services [0]	366.75 (+- 712.36)	246.94 (+- 577.45)	751.32 (+- 939.92)	<0.001
- Imaging [0]	188.97 (+- 325.13)	164.02 (+- 323.12)	269.05 (+- 321.14)	0.027
- Equipment [0]	159.74 (+- 224.07)	137.89 (+- 181.66)	229.88 (+- 316.73)	0.033
- Gases [0]	114.56 (+- 160.69)	98.89 (+- 130.28)	164.86 (+- 227.14)	0.033
- Food [0]	73.4 (+- 172.29)	67.62 (+- 176.51)	91.94 (+- 157.89)	0.306
- Per diem cost deprecated [0]	22.97 (+- 32.21)	19.83 (+- 26.12)	33.05 (+- 45.54)	0.033
- Endoscopy [0]	4.16 (+- 33.03)	0.95 (+- 4.44)	14.48 (+- 66.67)	0.116
Ward cost [0]	2947.75 (+- 3897.26)	3457.53 (+- 4191.26)	1311.52 (+- 2037.1)	<0.001
- Labs [0]	741.75 (+- 1008.6)	915.29 (+- 1083.16)	184.74 (+- 337.78)	<0.001
- Human resources [0]	654.23 (+- 867.59)	795.28 (+- 930.49)	201.5 (+- 353.16)	<0.001
- Materials [0]	563.01 (+- 872.19)	656.95 (+- 898.61)	261.48 (+- 707.2)	<0.001
- Drugs [0]	472.7 (+- 1114.32)	527.42 (+- 1168.3)	297.04 (+- 906.12)	0.107
- Dialysis [0]	136.14 (+- 1193.66)	168.91 (+- 1363.52)	30.97 (+- 153.5)	0.163
- Anatomic pathology [0]	126.49 (+- 86.76)	116.92 (+- 87.15)	157.21 (+- 78.55)	<0.001
- Imaging [0]	110.58 (+- 192.27)	122.9 (+- 200.8)	71.02 (+- 156.86)	0.036
- Food [0]	95.64 (+- 201.98)	114.32 (+- 224.5)	35.7 (+- 74.07)	<0.001
- Blood services [0]	45.47 (+- 171.77)	41.29 (+- 164.98)	58.88 (+- 192.78)	0.518
- Per diem cost depreciated [0]	20.41 (+- 27.07)	24.81 (+- 29.03)	6.29 (+- 11.02)	<0.001
- Endoscopy [0]	6.91 (+- 45.52)	7.08 (+- 45.46)	6.39 (+- 46.09)	0.918
- Equipment [0]	6.86 (+- 37.31)	8.92 (+- 42.53)	0.25 (+- 2.01)	0.005
- Gases [0]	2.38 (+- 31.63)	3.11 (+- 36.22)	0.03 (+- 0.18)	0.232
Total labs cost [0]	1444.81 (+- 1628.83)	1559.98 (+- 1766.12)	1075.17 (+- 1000.74)	0.007
OR average medical surgical center cost [0]	54.02 (+- 147.42)	63.99 (+- 158.54)	22.01 (+- 98.39)	0.013
